# Role of charge accumulation in guided streamer evolution in helium DBD plasma jets

**DOI:** 10.1038/s41598-021-96468-4

**Published:** 2021-08-26

**Authors:** Mikhail Pinchuk, Anton Nikiforov, Vadim Snetov, Zhaoquan Chen, Christophe Leys, Olga Stepanova

**Affiliations:** 1 Institute for Electrophysics and Electrical Power of the Russian Academy of Sciences, St. Petersburg, Russia 191186; 2 Department of Applied Physics, Ghent University, 9000 Ghent, Belgium; 3 School of Electrical and Information Engineering, Anhui University of Technology, Maanshan, 243032 China

**Keywords:** Electrical and electronic engineering, Applied physics, Plasma physics, Biomedical engineering

## Abstract

Experimental data are presented on the evolution of a helium atmospheric pressure plasma jet driven by a tailored voltage waveform generated as bunches of voltage pulses consisting of a superposition of $\approx 43$ kHz bipolar square pulses and $\approx 300$ kHz oscillations. The characteristics of directed ionization waves (guided streamers) are compared for bunches with different first pulse polarities and different bunch duty cycles. The longest and brightest streamers are achieved at the voltage bunch with the first negative pulse and a minimum duty cycle. The dynamics of streamers at the voltage bunch with the first positive pulse are characterized by the shortest length and a lower brightness. The plasma jet length can be smoothly changed by varying the number of pulses in the bunch and the polarity of the first pulse. It is thus possible to precisely localize the region of a strong field in space by combining the parameters of the applied voltage (the duty cycle and polarity of the first pulse of a bunch) with a stepwise propagation mode of a guided streamer.

## Introduction

Atmospheric pressure plasma jets (APPJs) have taken a significant place among the sources of nonequilibrium low-temperature plasma. Over the past two decades, an enormous amount of research has been focused on the study of cold APPJs. A significant number of extensive reviews^1–5^ and books^6–8^have also been published. This interest is due to the prospects of APPJ use in areas that are relevant for high demanding applications^9,10^, such as medicine, food technology, environmental protection, local surface modification, design of functional coatings and biotechnology. Several APPJ-based devices are certified for medicine and are being tested in clinical practice^8^. The main practical interest in APPJ is associated with the treatment of certain temperature-sensitive biological objects^11,12^ and the exploration of energy efficient modes of APPJ applications^13–16^.

The use of atmospheric pressure plasma jets is usually associated with the need to extend the plasma plume outside of an inter-electrode discharge gap. Usually, a dielectric barrier discharge (DBD) is ignited inside the discharge unit, through which a working gas flow is passed. The plasma jet is then formed as a guided streamer propagating from the exit of the discharge unit in the ambient air. This system is a plasma-chemical reactor, where the reactions occur not only inside the discharge unit, but also in the plasma jet expanding in the surrounding air. A stationary plasma jet visible to the eye is, in this case, the process of propagation of repeating ionization waves^17–19^ along the gas flow. It is detected via high-speed imaging as “plasma bullets” following each other^1,5,20^.

The head of the guided streamer is characterized by the maximum production of chemically active components in the jet region^21,22^. A high-field region in the head creates optimal conditions for the implementation of chemical reactions while mixing the ionized working gas and air. Due to this phenomenon, the plasma jet can effectively deliver chemically and biologically active species (oxygen and nitrogen reactive species) as well as excited high-energy particles to a region remote from the discharge unit.

Thus, controlling the propagation of a plasma jet provides a number of possible application advantages for the development of APPJ-based technologies and can help in design of adaptive cold atmospheric plasma sources^23^ for a variety of biomedical applications.

One of the approaches used to control the parameters of a plasma jet is the application of an external field to the system using additional electrodes at a certain bias potential or using a target with a bias potential^24–27^. It has been shown that an electric potential applied to the external additional electrode or processed target changes the direction and speed of propagation of a guided streamer.

However, it seems more technologically promising to control streamer propagation by using a special synthesized voltage signal. The method of controlling plasma characteristics by synthesizing a specially-shaped voltage signal is becoming a prominent research topic in the field of low-temperature nonequilibrium plasma^28^. The applied voltage shape variation changes the propagation dynamics of the guided streamer^25,29^. Earlier we recorded a stepwise propagation of a guided streamer for a helium APPJ with a supplied voltage consisting of a superposition of microsecond bipolar rectangular pulses and oscillating signals^30–32^. The possibility of controlled deceleration and acceleration of a guided streamer was also indicated in calculations^33^ performed for the modulated nanosecond pulses of applied voltage.

In general, plasma jets can be generated at many different voltage supply modes, for example, at a conventional sinusoidal one. They might operate for the very first voltage cycle and their occurrence does not require any preionization of a gas. But, the formation of a plasma bullet with high reproducibility and repeatability is possible only under certain parameters^5^. The process of forming a plasma jet begins to reproduce a certain time after the beginning of the voltage supply and from a certain minimum frequency and duration of the applied voltage^5^. This is associated with the need for the guided streamer development of a certain seed electron concentration of $\sim 10^{9}$ cm$^{-3}$, provided as the remaining background ionization from previous discharge cycle^5^. Additionally, after the initial switching ON of the voltage supply, the transition process from the irregular formation of the streamer to the regular formation (transitioning from the random mode to the repeatable mode, respectively) takes a certain amount of time^34^. It was indicated^34^ that the appearance time of the guided streamer at the first discharge pulses varies greatly from the beginning of the applied voltage pulses. On average, the APPJ length decreases sequentially for subsequent discharges for positive voltage pulses and reaches steady length after 3–5 subsequent discharges^34^. In work^29^, the largest positive streamer length was recorded after a large number of preliminary negative pulses.

In this work, we describe a scenario of a transition mode for guided streamer formation with a stepwise propagation, which can be used to precisely control the APPJ length. The mechanism is proposed to explain the formation of such plasma jet and its operational regime based on the accumulation of positively charged ions in space.

## Results

**Figure 1:**
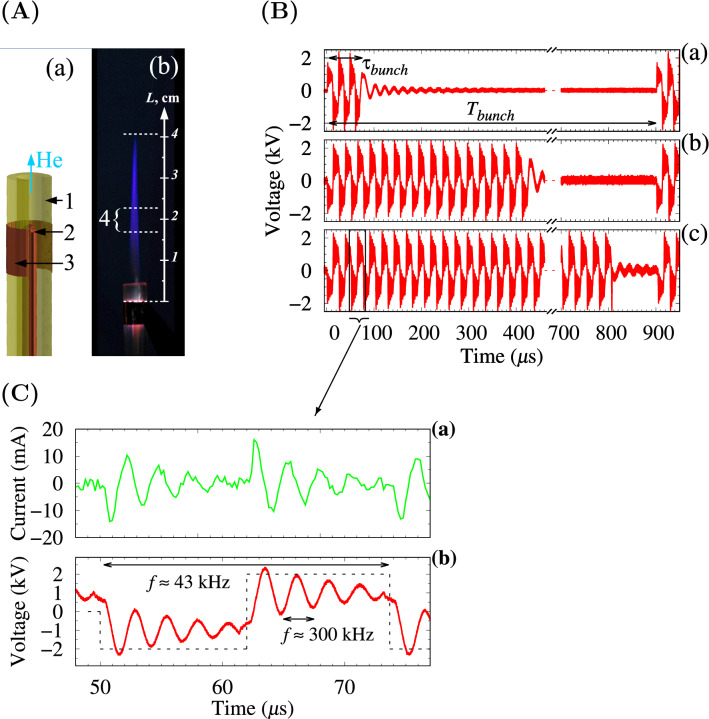
(**A**) Discharge unit (**a**) and photo of the plasma jet (**b**): 1—quartz tube, 2—high voltage rod electrode, 3—grounded foil electrode, 4—spectral intensity recording strip area of 6 mm width with centerline at 2.0 cm from the electrode edge; (**B**) Voltage signal across the discharge gap: (**a**) duty cycle of $\tau _{bunch} / T_{bunch} \approx 8$%, (**b**) 50% and (**c**) 90%; (**C**) Current (**a**) and voltage (**b**) signal across the discharge gap during a voltage pulse period.

The scheme of the discharge unit and a plasma jet photo are shown in Fig. 1A. The helium flow rate through the discharge tube was fixed at 6 l/min, at which the APPJ length has a maximum value with a laminar gas flow^35,36^. A more detailed description of the experimental setup can be found in the Methods section and in^37^.

The voltage signal was generated as bunches with variable duration $\tau _{bunch}$ and fixed repetition period $T_{bunch} = 900$ $\upmu$s. They were filled with a superposition of bipolar rectangular pulses with a frequency of $\approx 43$ kHz and an oscillating high voltage signal with a frequency of $\approx 300$ kHz. A voltage signal with duty cycles $\tau _{bunch} / T_{bunch}$ of $\approx 8$%, 50% and 90% and different polarities of the first pulse in a bunch was considered. To compare these modes, the experiments were performed at the same peak-to-peak voltage of 4.6 kV. The waveform of the voltage across the discharge unit for the three duty cycles is shown in Fig. 1B. The current curve and voltage signals across the discharge unit during one voltage period are shown in Fig. 1C. The time uniformity of the bunch repetition in the voltage signal for duty cycle of $\approx 8$% was evaluated using 50 bunches. The variation in their durations did not exceed 6 ns. It is necessary to emphasize that the above cases were investigated when the plasma jet has been generating for a long enough period of time (more than 5 min) before starting the measurements.

**Figure 2:**
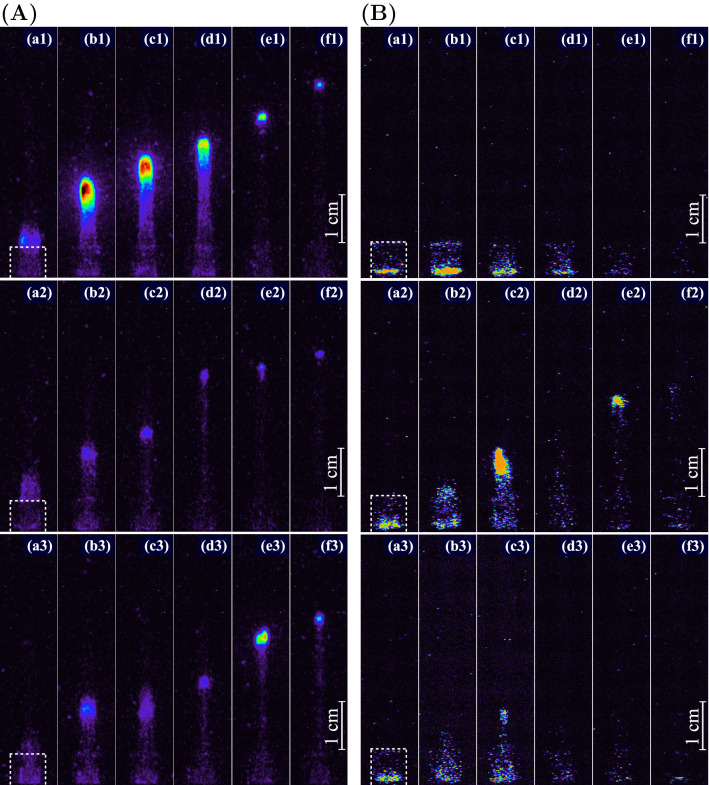
(**A**) ICCD images at the first three positive pulses for the first negative pulse in the bunch with duty cycle of $\approx 8$%: (**a1**) at 12.8, (**b1**) 13.1, (**c1**) 13.3, (**d1**) 13.5, (**e1**) 15.5 and (**f1**) 16.1 $\upmu$s; (**a2**) at 38.8, (**b2**) 39.1, (**c2**) 39.3, (**d2**) 39.5, (**e2**) 41.5 and (**f2**) 42.1 $\upmu$s; (**a3**) at 64.8, (**b3**) 65.1, (**c3**) 65.3, (**d3**) 65.5, (**e3**) 67.5 and (**f3**) 68.1 $\upmu$s. ICCD exposure time is of 50 ns with 50 accumulations. The dashed contours are the tube’s borders. The time of the images correspond to Fig. 3A. (**B**) ICCD images at the first positive pulse in the bunch: (**a1**) at 3.0, (**b1**) 3.6, (**c1**) 4.0, (**d1**) 5.4, (**e1**) 6.8 and (**f1**) 10.2 $\upmu$s; (**a2**) at 24.0, (**b2**) 24.6, (**c2**) 25.0, (**d2**) 26.4, (**e2**) 27.8 and (**f2**) 28.5 $\upmu$s; (**a3**) at 44.6, (**b3**) 45.2, (**c3**) 45.6, (**d3**) 47.0, (**e3**) 48.4 and (**f3**) 52.0 $\upmu$s. ICCD exposure time is of 50 ns with 5 accumulations. The dashed contours are the tube’s borders. The time of the images correspond to Fig. 3B.

**Figure 3:**
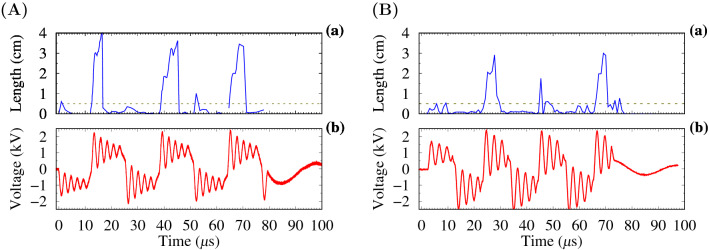
(**A**) APPJ length (**a**) and voltage signal (**b**) at the first negative pulse in the bunch with a duty cycle of $\approx 8$%. (**B**) APPJ length (**a**) and voltage signal (**b**) at the first positive pulse in the bunch. The horizontal dashed line shows the position of the tube orifice. The signals correspond to the images in Fig. 2.

At the positive pulses, the guided streamers of the longest length were formed. With a negative voltage polarity, a short streamer with a length of 2–3 mm was formed. It was revelated that the dynamics of ionization wave development depend on the pulse number from the beginning of the voltage bunch. High-speed ICCD images for the first three positive pulses at the first pulse of the negative (Fig. 2A) and positive (Fig. 2B) polarities for a duty cycle of 8% are shown in Fig. 2. The length of the streamers recorded synchronously with the voltage signal for these images is presented in Fig. 3.

The longest streamer was achieved in the first positive pulse at the voltage bunch starting with the negative pulse (Figs. 2A(a1–f1) and 3A). In addition, it was found that the first streamer in this case had the highest intensity. The dynamics of streamers in the bunch with the first positive pulse are characterized by a shorter length and a lower brightness (Figs. 2B(a1–f1) and 3B). The streamer at the first positive pulse was very short.

**Figure 4:**
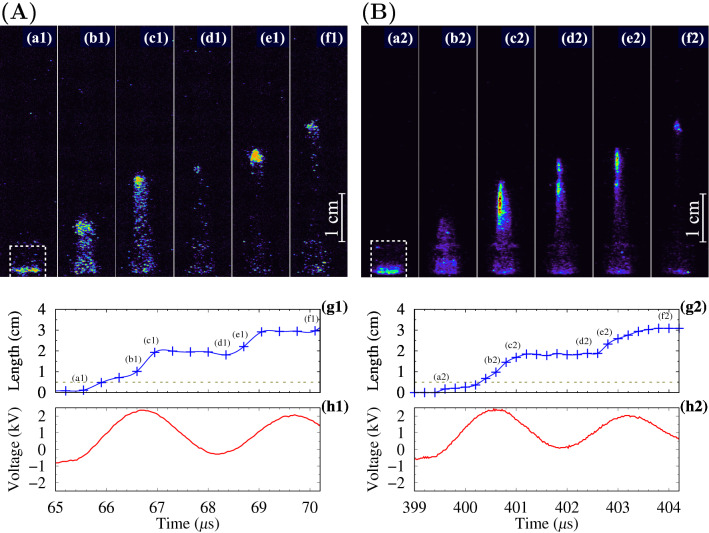
(**A**) High-speed ICCD images (**a1)–(f1**), APPJ length (**g1**) and voltage curve (**h1**) for the 4th positive pulse in the bunch with the first positive pulse as in Fig. 3B. (**B**) The 16th positive pulse in the bunch with the first negative pulse, as shown in Fig. 1B(b). The exposure for frames was 50 ns with 5 accumulations in (**a1**)–(**f1**) and 50 ns with 15 accumulations in (**a2**)–(**f2**). The labels in the frames (**a**)–(**f**) corresponds to position of points indicated in graphs (**g1**) and (**g2**). The dashed contours in (**a**) are the tube’s borders.

The settling of the streamer propagation dynamics occurs during these first three bipolar pulses. Starting from the third voltage period in the bunch, at each subsequent pulse, an APPJ is formed with good repeatability. High-speed ICCD images of the guided streamer development in the 4th positive pulse for a duty cycle of 8% and in the 16th pulse for a duty cycle of 50% are shown in Fig. 4. It is clearly seen from these images that there is a complete identity of the streamer development process in a stepwise propagation mode^30^, when the streamer’s motion has several stages: the first step of moving (see plot (g1) and (g2) between marks (a1–c1) and (a2–c2) in Fig. 4), a stop period (c1–d1 and c2–d2), and the second step of moving (d1–f2 and d2–f2).

The different numbers of signal accumulations for image averaging does not result in blurring of the corresponding images in Figs. 2 and 4. This indicates a very good reproducibility of the streamer formation in the plasma jet depend on the pulse number from the beginning of the voltage bunch.

**Figure 5:**
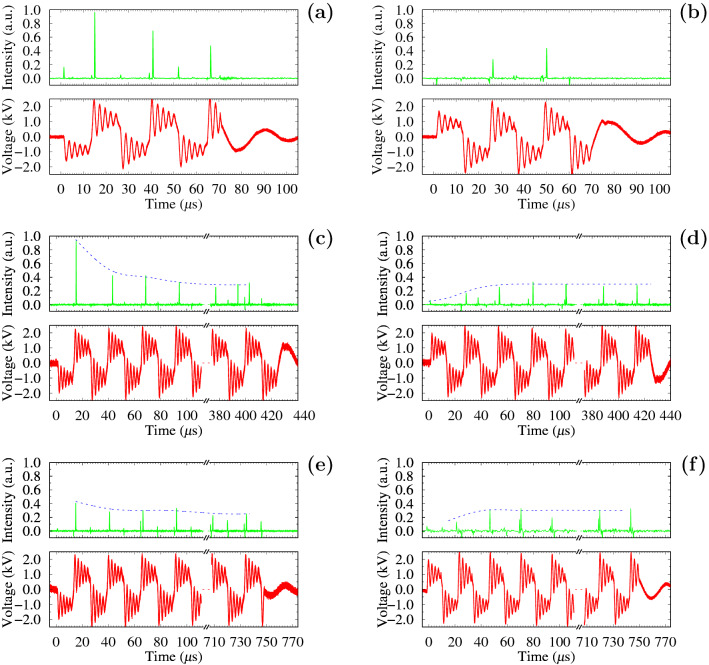
Light intensity at 706.5 nm (green lines) and voltage signal (red lines): (i) at the duty cycle of $\approx 8$% for the first negative (**a**) and positive (**b**) pulses in the bunch; (ii)  at the duty cycle of $\approx 50$% for the first negative (**c**) and positive (**d**) pulses in the bunch; (iii) at the duty cycle of $\approx 90$% for the first negative (**e**) and positive (**f**) pulses in the bunch. The signals were recorded with 50 accumulations. The time scale is chosen to represent one full bunch of the applied voltage.

The identity of the APPJ formation after the third positive pulse is confirmed by the signal from the radiation intensity of the jet. Emission intensities at a wavelength of 706.5 nm, corresponding to a helium line, at a distance of 1.5 cm from the tube edge (2 cm from the foil edge electrode) (see Fig. 1A(b)) for different duty cycles of the voltage signal are presented in Fig. 5. Radiation signals were observed in the same phase of the voltage pulses. At the duty cycle of 8%, they had different intensities in the bunch: 0.5–1.0—for the signal with the first pulse of negative polarity (Fig. 5a) and 0–0.5—for the signal with the first pulse of positive polarity (Fig. 5b). The highest intensity of the streamer was observed at the first positive pulse for the bunch started with the negative polarity. At the increase in the duty cycle to 50% (Fig. 5c, d) and 90% (Fig. 5e, f) the intensities of the radiation signals in the bunch became more comparable. This trend was observed independently of the first pulse polarity.

**Figure 6:**
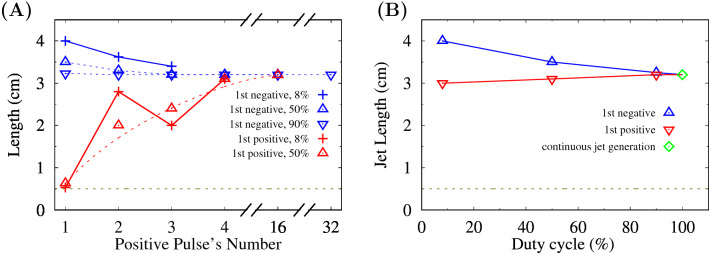
(**A**) The maximal length of the guided streamer versus the positive pulse number in a bunch for the first negative and first positive pulses. The 16th pulse is the last positive pulse for $\approx 50$% and the 32nd pulse is the last one for $\approx 90$%. (**B**) The maximal integral length of APPJ for different duty cylces. A point attributed to continius jet generation is from^32^.

The change in a streamer’s length corresponding to the positive pulse’s number in the bunch is presented in Fig. 6A. The negative polarity of the first pulse in the bunch gives us a longer streamer than the positive polarity in all the considered cases of the voltage signals. This is justified by the data on the integral length of the plasma jet (Fig. 6B). The increase in the duty cycle eliminates the difference between the plasma jet’s lengths at negative and positive polarities of the first pulse in the bunch.

## Discussion

A transient, but strictly regular process occurs during the full bunch cycle. The reasons for this behavior can be associated with the formation of a spatial pattern of uncompensated charges around the discharge region. Thus, there is a redistribution of the electric potential, which affects the formation and propagation development of the ionization waves^24,38,39^.

The formation of an uncompensated positive volume charge cloud for streamer discharges is a generally common case^39–41^. A charge flux of different polarity particles arises from the discharge area with a current closing on surrounding objects. However, it is rather difficult to measure the exact value of the charged particle flux into the surrounding volume from the discharge region and the value of the uncompensated volume charge outside the plasma jet region. During the voltage period there is no exact balance of the charge in the circuit, and there is a difference between the positive and negative charges in the circuit.

For example, the positive charge through the circuit at a positive voltage half-cycle is greater by $\approx 0.05$–0.1 nC than the negative charge at the negative voltage half-cycle from the statistical analysis^42^ for a surface dielectric-barrier discharge in air at a steady state regime, when a flowing charge in the circuit was estimated to be $\approx 0.1$–0.4 nC per voltage period. Because of the difference in charge generated in positive and negative half-period of the applied voltage, the charge starts to propagate from the discharge area into the surrounding space, forming a positive charge cloud around the discharge system^42^. The difference between the transfered positive and negative charges in the recent work^43^ was of the same order value for the helium plasma jet, which is similar to our configuration. The positive space charge formed in plasma jet^44,45^ is characterized by a volume charge of $\sim 1$ nC as found in simulations of the guided streamer development in the helium jet.

A charge of $\approx + 0.1$ nC in a $\sim 0.5$ cm diameter cloud has a potential of $\sim 1$ kV in the cloud center^46^ and the charge corresponds to a concentration of $\sim 10^{9}$ cm$^{-3}$. This potential can stop the streamer development^24^. On the other hand, a cloud charge less than 0.01 nC corresponds to a potential that is too low, which could not affect the streamer head with a field of $\sim 10$ kV/cm, which is a typical value of the E-field in APPJs^47^.

There is a rapid dispersion of an ionic cloud^46,48–50^ to the charge concentration close to the background value, which does not affect the dynamics of streamer propagation. In the case of a low degree of ionization ($< 10^{9}$ cm$^{-3}$ in our case, see Supplementary Information^46^), charged particles can be considered to be an independent component of the gas and it can be assumed that charged particles of each type diffuse through a neutral gas without a noticeable interaction between charged particles of the same or different type^48^. If the Debye radius $R_{D} = 4.86 (T_{e} \mathrm {[K]} /n_{e} \mathrm {[cm^{-3}]})^{1/2}$ [cm]^40^ is close to the radius of the cloud, then the attraction of the ions is not able to keep the electrons inside the cloud^50^, and the electrons leave the cloud during a time $\sim 10$ ns. The resulting ion cloud expands unders the force of Coulomb repulsion^46^ and the uncompensated charge is filled up during the following voltage cycle.

The diversity of many different types of long-living positive and negative ions is formed around the plasma jet^51–53^. Among them, the negative ions play a key role in the creation of seed electrons, which assist the initiation of the discharge^54^. Whereas the plasma jet dynamics are defined with the positive potential formed by the abundance of positive ions, mainly nitrogen ions, which compose the charged cloud. Indeed, the $\mathrm{N}_{2}^{+}$ FNS bands were of the highest intensity in our plasma^32^. $\mathrm{N}_{2}^{+}$ ions are likely to be produced from Penning ionization of nitrogen from helium metastables^2,55^. The characteristic expansion time of the nitrogen $\mathrm{N}_{2}^{+}$ ion cloud with a total charge of $+0.1$ nC and a concentration of $\approx 10^{9}$ cm$^{-3}$ is $\sim 100$ $\upmu$s (see Supplementary Information^46^). This estimation^46^ shows that a quasi-stationary balance of charge fluxes in space should be established by the time of an order of $\approx 100$ $\upmu$s.

**Figure 7:**
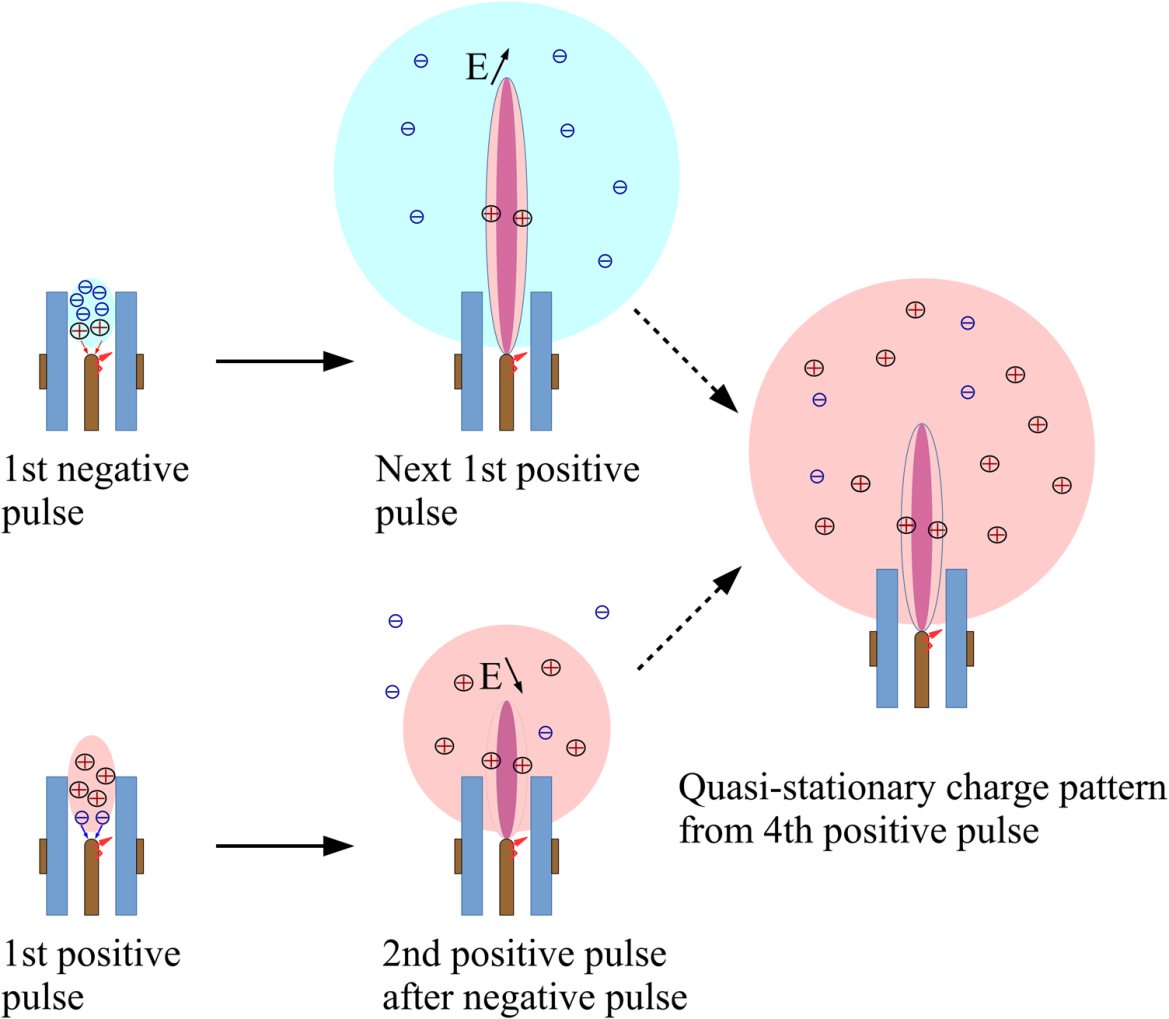
Outline of the APPJ evolution: formation of a charged cloud near the tube at the first discharge, further fast expansion of electrons and slower expansion of ions, and quasi-stationary positive charged cloud.

Thus, the possible mechanism was proposed for the interpretation of the observed effect of the voltage polarity and waveform on the jet evolution (see Fig. 7). The effect of voltage waveform and polarity is explained as follows. At the first negative pulse, a negative charge region is formed near the tube end, which enhances the field at the positive voltage front and supports the development of the next positive streamer of the longest length. This occurs insofar as the negative first pulse helps to draw out a positive charge from the discharge region and the negative charge is accumulated in the space. In contrast, at the first positive pulse a positive uncompensated charge is formed after the first discharge. The positive charge screens the field in the head of the propagating streamer at the positive voltage front. As a result, the streamer propagation is suppressed. The region of charge accumulation expands, and the potential distribution after the next streamer decay smooths down, which makes the formation of subsequent streamers less difficult.

Thus, during the first few periods of voltage in the bunch (on the order of one hundred microseconds), a quasi-stationary positive charge cloud was formed around the jet, which limits the propagation length of the plasma jet. Correspondingly, the polarity of the first pulse affected these first voltage periods. Further formation of the quasi-stationary charge pattern and smoothing of the potential distribution leads to a regularly reproduced process of streamer formation.

The time of the same order of one hundred microseconds was required for the relaxation of the uncompensated charge (see Supplementary Information^46^). For voltage signal with duty cycle of $\approx 90$% for the first negative polarity, the streamer generation in the first period differs only slightly from subsequent periods. However, at a duty cycle of 50%, i.e. with $\approx 450$ $\upmu$s pause between voltage bunches, the first three periods have very similar characteristics to the first three periods with the minimal considered duty cycle of $\approx 8$%.

The above-described qualitative picture (Fig. 7) is well illustrated by the radiation intensity at a helium line of 706.5 nm from the middle of the jet’s length (Fig. 5). As known the intensity of the helium lines is a strong function of the electron temperature in the range of 2–10 eV^56^. The electron temperature in the same range is a function of the reduced electric field^40^, and the emission intensity of the He I 706.5 nm line is directly related to the electric field strength^14,55^ because the main mechanism of He I excitation is the electron impact. It is clearly seen that the radiation intensity (Fig. 5) in pulses decreases with an increase in the duty cycle, which reflects the field decrease in the head of the propagating streamer.

## Summary

Based on the presented results a novel way to control APPJ intensity and propagation length is proposed. By changing the number of pulses in the bunch (the duty cycle) and using the variable polarity of the first pulse, the plasma jet length can be smoothly adjusted (Fig. 6B). It is possible to precisely localize the region of a strong electric field in space through the combination of the parameters of applied voltage waveform with a mode of stepwise propagation of the streamer.

## Methods

### Atmospheric pressure plasma jet

An APPJ was directed vertically upwards. A discharge configuration of an “inner central rode—gas gap—quartz tube (dielectric barrier)—outer ring” was used. A high voltage was applied to the copper inner electrode, which had a diameter of 1.5 mm. The outer grounded electrode was made from 5-mm-thick copper foil wrapped 5 mm from the tube orifice. The discharge unit was located 20 cm above the grounded metal surface of the optical table. The distance to all of the other objects was more than 0.5 m. The plasma jet had been generated for more than 5 min before the measurements.

**Figure 8:**
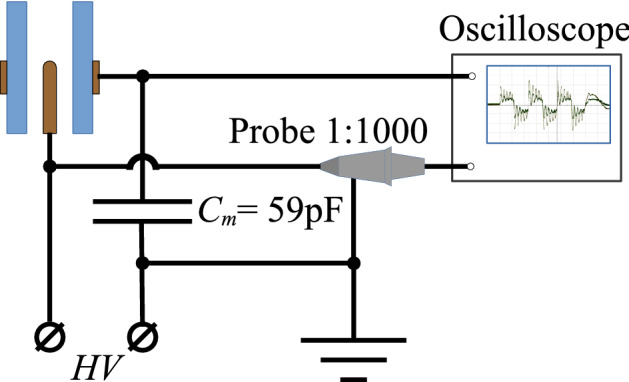
Electrical measurement scheme: *HV*—applied high-voltage signal; $C_{m}$—measuring capacitance; Probe 1:1000 is a high-voltage divider.

The electrical measurement scheme is shown in Fig. 8. The voltage signals across the discharge unit for the three duty cycles are shown in Fig. 1B. The current and voltage across the discharge unit during the voltage pulse period are shown in Fig. 1C. The voltage across the gas discharge unit was measured with a Tektronix P6015A high voltage divider. The charge flowing through the discharge unit was measured by means of the capacitance of 59 pF. All signals were recorded with an oscilloscope (LeCroy WS64Xs, Teledyne LeCroy, USA, 600 MHz bandwidth or Tektronix TDS 2024C, USA, 200 MHz bandwidth). The current was obtained as a time derivative of the recorded charge waveform function.

The helium flow rate was controlled with a Mass-Flo 1179 gas flow controller (MKS Instruments, USA). The gas flow rate was maintained at 6 l/min, at which the APPJ length has a maximum value with a laminar gas flow^35,36^. The gas flow rate of 6 l/min corresponds to a laminar flow with a Reynolds number of $\approx 270$. The quartz tube had an internal diameter of 4.6 mm and a wall thickness of 1 mm. Additional details of the experimental system can be found in the paper^37^.

### High speed imaging

The guided streamer propagation along the APPJ into the ambient air was recorded by means of an intensified charge-coupled device (ICCD) camera Andor iStar DH340T (Oxford Instruments, UK; pixels: $2048 \times 512$, minimum optical gate: 1.9 ns, frame camera speed $\sim 1$ Hz) and a Hamamatsu C8484-05G charge-coupled device camera with a W7571-01 image intensifier unit (Hamamatsu Photonics K.K., Japan; pixels: $1024 \times 1024$, minimum optical gate: 5 ns, frame camera speed $\sim 10$ Hz). The frame exposure was of 50 ns with different accumulation numbers from 1 to 50 depending on the emission intensity. High-speed imaging was conducted using the time strobing method, with each new accumulation produced during a new voltage bunch with a certain offset relative to the reference trigger time.

### Spectral intensity recording

The emission signal at a wavelength of 705.6 nm was recorded using a photomultiplier tube R928 (Hamamatsu Photonics K.K., Japan) connected to an Omni $\lambda$750 spectrograph (Zolix Instruments Co., LTD, Beijing, China; with a grating of 1200 grooves/mm, blazed on 500 nm) via the LeCroy WS64Xs (Teledyne LeCroy, USA; bandpass 600 MHz) oscilloscope at full bandwidth with 50 signal accumulations. Fiber optics of $100\, \upmu$m diameter with the collimating lens has been connected to the spectrometer. The light collection was taken from a strip area 6 mm wide with a centerline 2.0 cm from the foil edge electrode perpendicular to the jet axis (see Fig. 1A(b)).

### Plasma jet length determination

The jet length was determined by processing the ICCD images with a Python script. To exclude noise typical for cameras with image intensifiers, fast smoothing was performed using the spatial Fourier transform with a cutoff from considering the disconnected areas in the images with diameters less than 0.5 mm. The position of the ionization front was taken as the position farthest from the edge of the foil grounded electrode, with a brightness exceeding $4 \sigma$ of the background brightness, where $\sigma$ was the standard deviation of the background brightness in the image part that does not contain a jet radiation signal. The jet brightness varied greatly in the different voltage periods (Fig. 5). Such a criterion for the brightness threshold made it possible to compare the behavior of the jet with a strong difference in brightness, depending on the parameters under study.

## Supplementary Information

Supplementary Information.
