# Supplementary material for: Role of charge accumulation in guided streamer evolution in helium DBD plasma jets
Source: Sci Rep. 2021 Aug 26;11:17286. doi: 10.1038/s41598-021-96468-4 (PMC8390516; doi:10.1038/s41598-021-96468-4)
Supplement: Supplementary file 1 — Supplementary Information. [file 41598_2021_96468_MOESM1_ESM.pdf]

Supplementary Information for the manuscript Role of charge accumulation in guided streamer evolution in helium DBD plasma jets

Mikhail Pinchuk[1,*], Anton Nikiforov[2], Vadim Snetov[1], Zhaoquan Chen[3], Christophe Leys[2], and Olga Stepanova[1]

[1]Institute for Electrophysics and Electrical Power of the Russian Academy of Sciences, St. Petersburg, 191186, Russia
[2]Ghent University, Department of Applied Physics, Gent, 9000, Belgium
[3]Anhui University of Technology, School of Electrical and Information Engineering, Maanshan 243032, China
[*]pinchme@mail.ru

This Supplementary Information contains an estimation of the charged cloud expansion consisting of cold ions.

Consider the expansion of a spherically symmetric charged homogeneous cloud of cold ions with initial radius R_0 in a gas as a result of mutual charge-charge repulsion (mutual Coulomb repulsion). Let us assume that the uniformity of the sphere is preserved during expansion relative to the sphere center. Additionally, assume that the Debye radius is close to the radius of the cloud. Then, the attraction of the ions can not keep the electrons inside the cloud[1], and the electrons will leave the cloud when the time $\sim R_0/v_e < 10$ ns. The resulting ion cloud without electrons will expand because of the force of Coulomb repulsion.

In the case of a low degree of ionization, charged particles can be considered as an independent component of the gas, and it can be assumed that charged particles of each type diffuse through a neutral gas without noticeable interaction between each other or charged particles of other types[2]. The Coulomb repulsion in addition to diffusion must be considered[2] starting at an ion concentration of 10^5 cm^{-3}. Coulomb mutual repulsion prevails over diffusion at an ion concentration above 10^6 cm^{-3}. At a concentration above $\sim 10^9$ cm^{-3}, the Debye radius $R_D = 4.86(T_e[\mathrm{K}]/\mathrm{n_e}[\mathrm{cm}^{-3}])^{1/2}$ [cm] size[3] becomes much smaller than the system, and this model does not describe the system well[3].

In the vicinity of streamer discharges in air, the concentration of uncompensated charges[3–5] is estimated to be at a level of 10^8 cm^{-3}. For a helium plasma jet flowing into the air, an uncompensated charge[6] on the order of this value forms near the discharge tube exit. Thus, it is reasonable to consider the expansion of a cloud with charges Q of 0.001, 0.01, 0.1 and 1 nC and a radius of $R_0 = 0.5$ cm. These charges Q of 0.001, 0.01, 0.1 and 1 nC correspond to concentrations n_+ of $\approx 10^7$, 10^8, 10^9 and 10^{10} cm^{-3}, respectively.

The charged particle drift velocity at cloud radius $v_+ = \mu_+ E$ is equal to the cloud border expansion velocity, where $E = 3Q/(8\pi\varepsilon_0 R)$ is the field at the cloud radius, $Q = (4\pi/3)en_{0+}R_0^3$ is the full cloud charge, $R = (3Q/(4\pi e n_+))^{1/3}$ is the cloud radius, e is the elementary charge, and μ_+ is the ion mobility.

Let us consider two cases, one for helium ions He^+ and another one for nitrogen ions N_2^+. Helium ions He^+ have the highest mobility, and ions of other types such as $N^+, N_3^+, N_4^+, NO^+, O_2^+$ have mobilities close to the mobility of the chosen nitrogen ion, N_2^+. Therefore, these two cases can fully and qualitatively characterize our system. The ion mobility can be considered as constant[2] for the condition and takes the following values[2,7] in our estimations: $\mu_+ = 10$ cm^2/V·s for He^+ and 2 cm^2/V·s for N_2^+.

The problem is formulated by solving a differential equation for the ionic cloud border motion[3]:

$$\frac{\mathrm{dR}}{\mathrm{dt}} = \mu_+ E. \tag{1}$$

Equation (1) is equivalent to the following equation:

$$\frac{\mathrm{dn}_+}{\mathrm{dt}} = \frac{e\mu_+}{\varepsilon_0} n_+^2. \tag{2}$$

The solution to the equation (2) is as follows:

$$\frac{1}{n_+} - \frac{1}{n_{0+}} = \frac{e\mu_+}{\varepsilon_0} t. \tag{3}$$

The electric potential φ_c at the center of the cloud will be $\varphi_c = en_+R^2/(2\varepsilon_0)$.

The decrease in time of the concentration n_+ for the two ion types and the potential φ_c in the center of the ion cloud are shown in Figure S1.

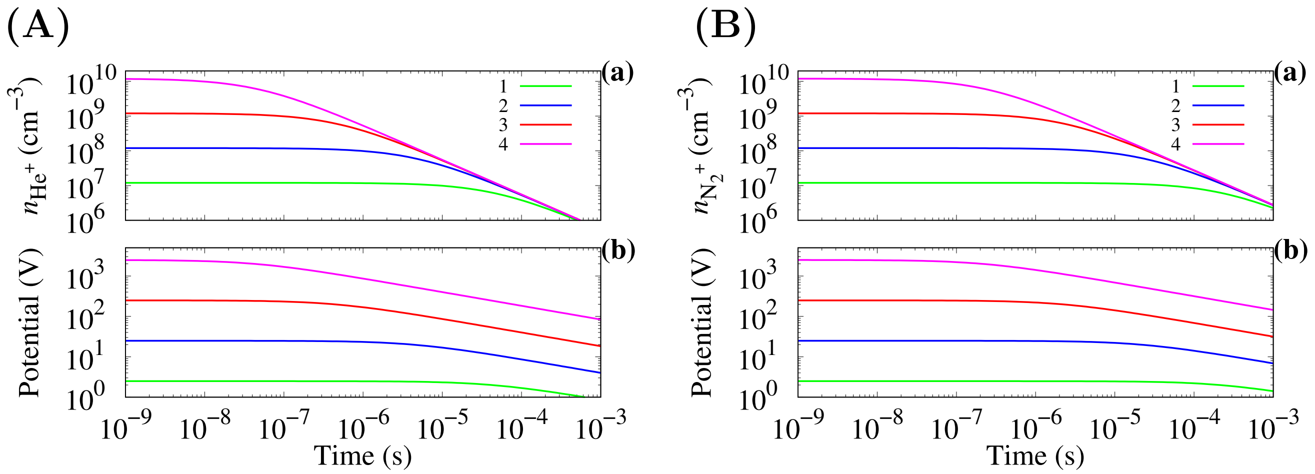

Figure S1. Dispersion of ionic cloud. **(A)** Concentration of ions He^+ in the cloud **(a)** and potential in the center of the cloud **(b)** with initial charges Q of 0.001 **(1)**, 0.01 **(2)**, 0.1 **(3)** and 1 nC **(4)** with a cloud radius of $R_0 = 0.5$ cm. **(B)** Concentration of ions N_2^+ in the cloud **(a)** and potential in the center of the cloud **(b)**.

Current model does not include the effect of negative ions presence in the vicinity of the cloud. However, it is logical to consider that the main influence on the formation of the plasma jet regime is caused by the formation of a positive uncompensated charge cloud. The source of ions determined by He electron impact ionization and the ionization of nitrogen through the metastable states of helium atoms, which experience only slow diffusion.

A significant decrease in the potential and concentration occurs within a time of the order of tens of microseconds. In this case, various positive nitrogen ions will determine the final quasi-stationary distribution of the charge and potential in the system. This time value corresponds to the observed time at which the repetitive regime of the plasma jet formation is established and vice versa, as well as the relaxation time after the end of the voltage bunch.

References

1. Gintsburg, M. A. Dispersion of ionic cloud. *Doklady Akademii Nauk USSR* **215**, 1337–1340 (1974).
2. McDaniel, E. *Collision Phenomena in Ionized Gases*. Wiley series in plasma physics (Wiley, 1964).
3. Raizer, Y. P. *Gas Discharge Physics* (Springer-Verlag Berlin Heidelberg, 1991).
4. Bazelian, E. M. & Raizer, Y. P. *Spark Discharge* (CRC Press, 1998).
5. Bazelyan, E. M. & Raĭzer, Y. P. The mechanism of lightning attraction and the problem of lightning initiation by lasers. *Physics-Uspekhi* **43**, 701–716, DOI: 10.1070/pu2000v043n07abeh000768 (2000).
6. Zhu, P., Li, B., Duan, Z. & Ouyang, J. Development from dielectric barrier discharge to atmospheric pressure plasma jet in helium: experiment and fluid modeling. *J. Phys. D: Appl. Phys.* **51**, 405202, DOI: 10.1088/1361-6463/aadb12 (2018).
7. McDaniel, E. & Mason, E. *The Mobility and Diffusion of Ions in Gases*. Wiley series in plasma physics (Wiley, 1973).
